# Atypical primary hyperparathyroidism due to parathyroid lipoadenoma: a case report

**DOI:** 10.1093/jscr/rjab308

**Published:** 2021-07-23

**Authors:** Francesco Cammarata, Al’ona Yakushkina, Luca Pennacchi, Luca Carsana, Pietro Zerbi, Giulio Montecamozzo, Piergiorgio Danelli

**Affiliations:** Department of General Surgery, Luigi Sacco University Hospital, Milan, Italy; Department of General Surgery, Ospedale di Saronno, Saronno VA, Italy; Department of General Surgery, Luigi Sacco University Hospital, Milan, Italy; Department of Pathology, Luigi Sacco University Hospital, Milan, Italy; Department of Pathology, Luigi Sacco University Hospital, Milan, Italy; Department of Biomedical and Clinical Sciences Luigi Sacco, University of Milan, Milan, Italy; Department of General Surgery, Luigi Sacco University Hospital, Milan, Italy; Department of General Surgery, Luigi Sacco University Hospital, Milan, Italy; Department of Biomedical and Clinical Sciences Luigi Sacco, University of Milan, Milan, Italy

## Abstract

Parathyroid lipoadenoma is a very rare cause of primary hyperparathyroidism. Preoperative imaging techniques often fail to detect such lesions, and even during surgery they can be misinterpreted just as fat tissue. A 62-year-old woman clinically monitored for primary hyperparathyroidism, with hypertension and a left nephrectomy for hydrouretheronephrosis caused by recurrent kidney stones. A neck ultrasound showed a nodule consistent with left parathyroid of 9 × 5 mm, which was not confirmed on single-photon-emission computed tomography/computed tomography (CT) scan. On surgery, a voluminous lesion with adipose appearance and texture was removed. Frozen sections and intraoperative parathyroid hormone (PTH) confirmed such lesion to be a parathyroid lipoadenoma. Parathyroid lipoadenomas are difficult to localize preoperatively. Sometimes they can be seen by ultrasound scan as hyperechoich lesion, but scintigraphy and CT often fail to identify them. Only the awareness of such lesions and the use of intraoperative PTH can avoid unnecessary extensive cervical exploration.

## INTRODUCTION

The most common cause of primary hyperparathyroidism is a single parathyroid adenoma in >80–85% of cases; less common are parathyroid hyperplasia, parathyroid carcinoma and multiple adenomas in more than one gland [1]. Parathyroid lipoadenoma is a very rare cause of primary hyperparathyroidism, which can cause difficulties in the diagnostic and therapeutic management [2, 3]. Due to the high adipose tissue content, preoperative imaging techniques often fail to detect lipoadenomas [2, 4, 5]. Sometimes it can be seen as a hyperechoic lesion on ultrasound or localized as lipoma-resembling lump on computed tomography (CT), although usually only retrospectively. Due to its rarity and peculiar presentation on imaging, surgery and histology, only the awareness of the possibility of this lesion and the use of intraoperative parathyroid hormone (PTH) assay allows to achieve a diagnosis and prevent an unnecessarily extended cervical bilateral exploration or a surgical treatment failure.

## PATIENT

A 62-year-old woman was clinically monitored for primary hyperparathyroidism since 2005. She had a medical history of arterial hypertension and in 2015 she underwent a laparotomic left nephrectomy for hydroureteronephrosis caused by recurrent kidney stones.

In March 2017, she had a neck ultrasound showing a nodule with a size of 9 × 5 mm in the lower third of the left thyroid lobe, consistent with left parathyroid. Subsequent T99m single-photon-emission computed tomography (SPECT)/CT scans did not show any uptake of the radioactive tracer at the level of the lower left thyroid gland different from the surrounding glandular tissue. A left parathyroidectomy was scheduled and preoperative laboratory tests showed elevated PTH at 453 pg/ml, low Vitamin D at 25.1 ng/ml, elevated serum calcium of 12.9 mg/dl, slightly reduced level of phosphorus. A neck CT was performed, not showing solid growing mass in the cervical district, in the absence of observable thyroid or parathyroid lesions.

**Figure 1:**
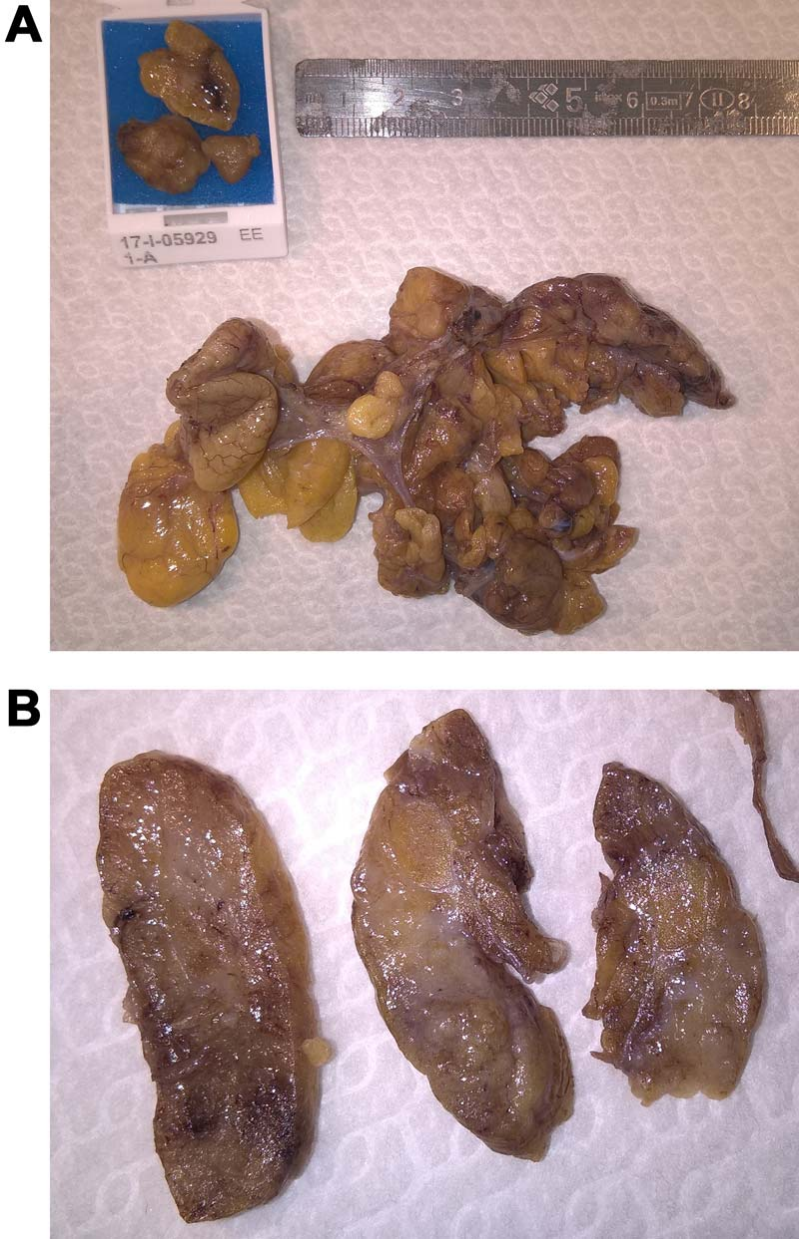
Surgical specimen showing a voluminous multilobulated with adipose appearance and texture, with a size of 7 × 7 cm.

Intraoperative PTH at the beginning of surgery was 440 pg/ml. The intervention started with a Kocher cervicotomy and cervical exploration at the left lower thyroid lobe. Here, there was a voluminous lesion with adipose appearance and texture. After identification and preservation of recurrent laryngeal nerve, the lesion was completely excised. On macroscopic investigation it was multilobulated, with a size of 7 × 7 cm, containing a little and poorly defined lump consistent with a pathologic parathyroid (Fig. 1). The sample was sent to obtain frozen sections and definitive histological examination. Frozen sections revealed the presence of adipose and connective tissue with tubular epithelial basaloid proliferation made of little cells without significant nuclear atypia, with focal areas of possible oncocytic differentiation. In those sections, the morphological features did not allow to distinguish between a thymic or parathyroid origin of the proliferation. After the excision intraoperative PTH dropped down to 34 pg/ml, supporting the decision not to proceed with bilateral cervical exploration.

**Figure 2:**
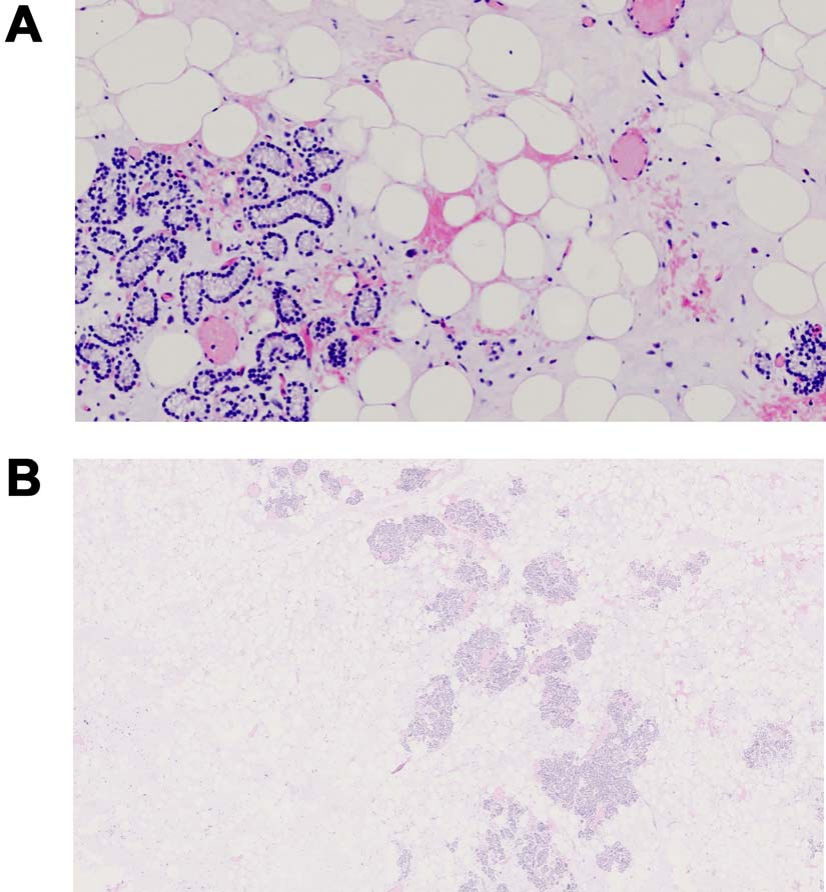
Pathological examination characterized by significant presence of fatty tissue with inside a cord proliferations of main and oncocytic cells, with sclerosis phenomena, and of lobular structures consisting of solid, cordal and microfollicular proliferations of parathyroid cells mixed with variable amounts of adipocytes.

The final pathology report described that in all sections examined there was a remarkable presence of fatty tissue with inside a cord proliferations of main and oncocytic cells zonally associated with sclerosis phenomena, and of lobular structures consisting of solid, cordal and microfollicular proliferations of parathyroid cells mixed with variable amounts of adipocytes (Fig. 2). Parathyroid cells, positive for CK-pan chromogranin A (80%), are zonally reactive for CD57 (<5%), in the absence of reactivity for calcitonin, synaptophysin and CD56. Ki67 < 2% (focal). Not histologically provable thymic structures. The histological picture and the immunohistochemical profile guided for a diagnosis of parathyroid lipoadenoma.

The patient was discharged on the third postoperative day in good general conditions. At discharge the patient’s serum calcium was 9.7 mg/dl and PTH was 12 pg/ml.

## DISCUSSION

Parathyroid lipoadenoma is a rare cause of primary hyperthyroidism, with <80 cases described in literature, comprising only case series and very few reviews [2, 3]. Due to the lack of extensive studies focused on this quite peculiar disease, it is relevant to take into account this unusual presentation that could lead to difficulties in the diagnostic process and also in a challenging identification during the surgical intervention if one does not consider the possibility of a lipoadenoma.

A major problem about parathyroid lipoadenomas is to localize them with commonly used imaging modalities such as ultrasound, CT and scintigraphy [2, 4].

This is caused by the fact that lipoadenomas, contrary to typical parathyroid adenoma, have an abundant fatty stroma that is seen hyperechoic on ultrasound and therefore difficult to detect.

SPECT reported a sensitivity of 89–95% in typical parathyroid proliferations, but in parathyroid lipoadenomas sensitivity is markedly decreased because of low concentration of parathyroid cells and the excess amount of adipose stroma. The presence of excess fat does indeed change the imaging characteristics of this type of adenoma, making them more difficult to localize [5].

Cervical exploration could fail to identify an enlarged parathyroid gland because of the abundant adipose tissue around. The rarity of this lesion makes it difficult to recognize and suspect intraoperatively.

A condition that goes in differential diagnosis with lipoadenomas is lipohyperplasia, in which lipomatosis and enlargement are found in several or all parathyroid glands [6]. Also a parathyroid carcinoma infiltrating the adipose tissue may be mistaken to a lipoadenoma, underlying the need of a careful pathologic examination, given the very different clinical significance of such differential diagnosis.

Since lipoadenomas can mimic normal parathyroid tissue at histology, pathologists need to be aware of this lesion especially during intraoperative sections. A conclusive parathyroid vs stromal fat cells ratio to distinguish lipoadenomas from normal glands is not yet defined, although some authors reported using fat cells percentages cut-offs ranging from 30 to 50% [7, 8]. Therefore, a definitive diagnosis of functioning parathyroid lipoadenoma usually comprises the pathologic diagnosis of a parathyroid proliferation within a fatty stroma combined with a significant intraoperative PTH drop after the excision [3]. However, the possibility of a non-functioning parathyroid lipoadenoma or a coexisting adenoma of another gland should be considered.

As in this case we described, the intraoperative dosage of the PTH is very useful for discriminating the cause of hyperparathyroidism, avoiding an unnecessary and possibly dangerous more extensive cervical exploration and discriminating between lipoadenoma and lipohyperplasia, avoiding the possible persistence or recurrence of disease.
